# Can Propolis Be a Useful Adjuvant in Brain and Neurological Disorders and Injuries? A Systematic Scoping Review of the Latest Experimental Evidence

**DOI:** 10.3390/biomedicines9091227

**Published:** 2021-09-15

**Authors:** Felix Zulhendri, Conrad O. Perera, Steven Tandean

**Affiliations:** 1 Kebun Efi, Kabanjahe 22171, North Sumatra, Indonesia; 2 Food Science Program, School of Chemical Sciences, University of Auckland, 23 Symonds Street, Auckland CBD, Auckland 1010, New Zealand; c.perera@auckland.ac.nz; 3 Department of Neurosurgery, Faculty of Medicine, Universitas Sumatera Utara, Medan 20222, Sumatera Utara, Indonesia

**Keywords:** adjuvant therapy, brain, neurology, nutraceuticals, pharmaceuticals, propolis

## Abstract

Propolis has been used therapeutically for centuries. In recent years, research has demonstrated its efficacy as a potential raw material for pharmaceuticals and nutraceuticals. The aim of the present scoping review is to examine the latest experimental evidence regarding the potential use of propolis in protecting the brain and treating neurological disorders and injuries. A systematic scoping review methodology was implemented. Identification of the research themes and knowledge gap was performed. After applying the exclusion criteria, a total of 66 research publications were identified and retrieved from Scopus, Web of Science, Pubmed, and Google Scholar. Several key themes where propolis is potentially useful were subsequently identified, namely detoxification, neuroinflammation, ischemia/ischemia-reperfusion injury/traumatic brain injury, Alzheimer’s disease, Parkinson’s disease, and epilepsy models, depression, cytotoxicity, cognitive improvement, regenerative medicine, brain infection, and adverse effects. In conclusion, propolis is shown to have protective and therapeutic benefits in alleviating symptoms of brain and neurological disorders and injuries, demonstrated by various in vitro studies, animal models, and human clinical trials. Further clinical research into this area is needed.

## 1. Introduction

Propolis is a natural, non-toxic, and resinous substance collected by bees to maintain hive homeostasis and to provide physical and biochemical protection to the hive [1,2,3]. Propolis has been used therapeutically for centuries as it possesses various biological activities including antimicrobial, anti-inflammatory, anti-cancer, and antioxidant properties [4,5,6]. Several preliminary clinical studies have also demonstrated the efficacy of propolis as an adjuvant for treating Sars Cov-2 infections [7,8]. Therefore, propolis appears to be a promising raw material for the future development of new therapeutic compounds. The biological activities and therapeutic properties of propolis are shown to be due to its content of plant secondary metabolite compounds such as phenolics and terpenoids [9].

Another exciting area of research is the use of propolis in treating neurological and brain disorders. However, the efficacy of propolis in this particular area has not been thoroughly explored. The main objectives of the present scoping review are to investigate the landscape of propolis research, identify the knowledge and research gap, and provide guidance for future research investigating the potential therapeutic uses of propolis in treating brain and neurological injuries, either as pharmaceuticals or nutraceuticals.

## 2. Methods

The scoping review was performed in accordance to the guidelines provided by Peters et al. and Munn et al. [10,11]. The four-phase flow diagram of the Preferred Reporting Items for Systematic Reviews and Meta-Analyses (PRISMA) was followed [12].

### 2.1. Search Strategy and Study Selection

The guiding question for the present scoping review was as follows: Can propolis be used as adjuvant therapy and/or to protect brain and treat neurological disorders? Two independent reviewers (F.Z. and S.T.) performed the searches up to 5 August 2021. The databases searched were Scopus, Pubmed, Web of Science, and Google Scholar. Table S1 illustrates the search strategy and the terms included in the search. Limited keyword searches were used for Google Scholar as expansive keyword searches appeared to be redundant.

The objective of the present scoping review is to evaluate the latest experimental evidence in the potential use of propolis, and therefore the search was limited to the last ten years of research: 2012–2021. In addition, we also focus on the studies that evaluate propolis as a whole and not the individual bioactive components of propolis. Consequently, we did not use the terms that describe individual bioactive components of propolis such as caffeic acid phenethyl ester (CAPE), pinocembrin, apigenin, and so on. However, during the search process, if the articles that described the individual bioactive components of propolis appeared, we would include them in the screening process. Moreover, we excluded studies that use synthetic derivatives of propolis bioactive compounds.

### 2.2. Eligibility Criteria

Any article that describes the potential use of propolis in protecting the brain or treating neurological disorders was selected including in vitro studies, animal models, and human clinical trials. We included all articles from all fields of science and technology. The titles and abstracts were analyzed and selected according to the eligibility criteria. Review studies were excluded as they might impart biases to the present study. Only articles that were written in English were included.

### 2.3. Data Collection

Two reviewers (F.Z. and S.T.) assessed the search results independently. If any disagreement arose on the eligibility criteria of a particular article, the disagreement was resolved through discussion and consensus. The studies that were both included and excluded were recorded in Mendeley. The duplicates were then removed. The collected articles were then screened by analyzing the titles, keywords, and abstracts. The articles that did not fit in the guiding question were subsequently removed. For the remaining articles, further screening was performed by analyzing the full texts. For articles where we could not find the full text, we analyzed the abstracts and subsequently included the articles if the abstracts clearly stated the experimental methods, analyses, and detailed results.

The following data of the resulting articles were then collected and tabulated in Microsoft Excel; full reference, types of study, types of propolis extract and/or propolis bioactive compounds, geographic locations of the propolis source, and measured outcome. The reviewers subsequently analyzed the titles, abstracts, and full texts and categorized the included studies into the appropriate themes.

## 3. Results

**Figure 1:**
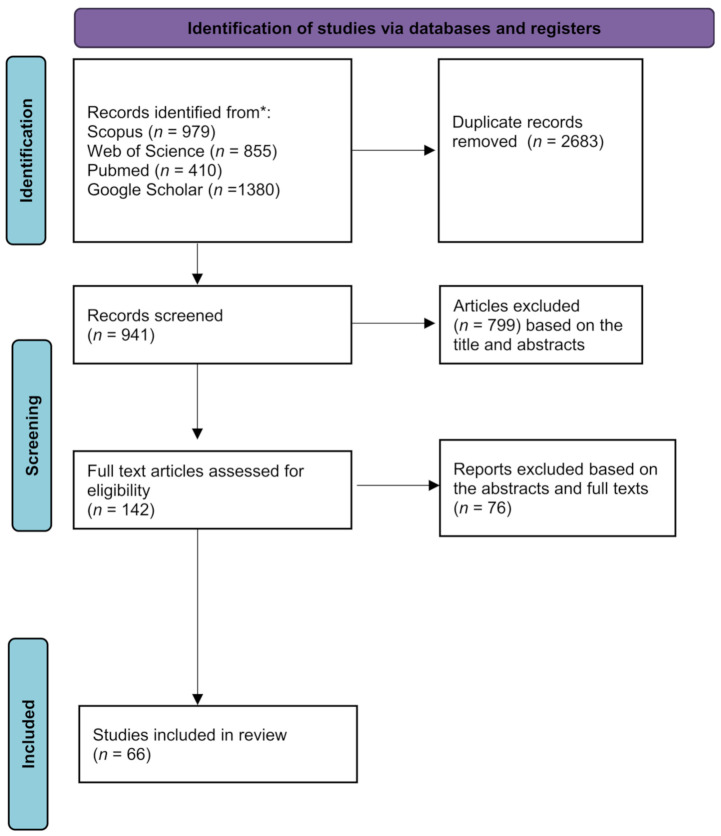
The screening process of the studies adapted from Preferred Reporting Items for Systematic reviews and Meta-Analyses (PRISMA).

The initial search resulted in 3624 scientific articles. Duplicates of 2683 were subsequently removed. Further screening based on the titles and abstracts excluded 799 articles. The full texts of the 142 articles were then analyzed and screened. The final screening resulted in 66 articles. Figure 1 illustrates the screening process.

**Figure 2:**
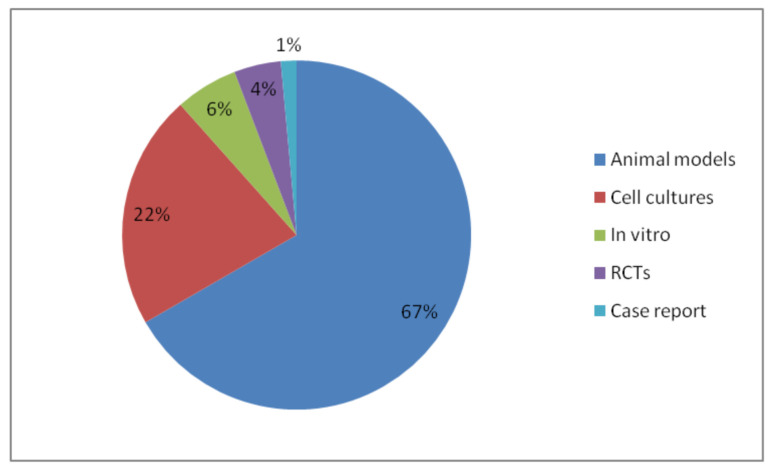
The percentages of the types of studies investigating the therapeutic use of propolis in the brain and in neurological disorders and injuries.

The qualitative analysis of the 66 included articles was performed and the articles were categorized into several themes. Table 1 illustrates the themes represented in the included studies; brain infection (2%), ischemia/ischemia-reperfusion/traumatic brain injuries/aneurysm (14%), detoxification (24%), Alzheimer’s disease model (9%), Parkinson’s disease model (8%), epilepsy model (3%), cognitive improvement (6%), regenerative medicine (3%), neuroinflammation/pain/oxidative stress (12%), depression & stress models (9%), cytotoxicity (8%), adverse effects (2%), and others (2%). Table 2 summarizes the themes, references, and the main findings alongside the types of propolis extracts and bioactive compounds used in the included studies. In addition, Figure 2 illustrates the types of studies in the included articles, namely animal models (67%), cell cultures (22%), in vitro (6%), randomized placebo-controlled human clinical trials (4%), and case reports (1%). The percentages are rounded to the nearest whole number.

**Figure 3:**
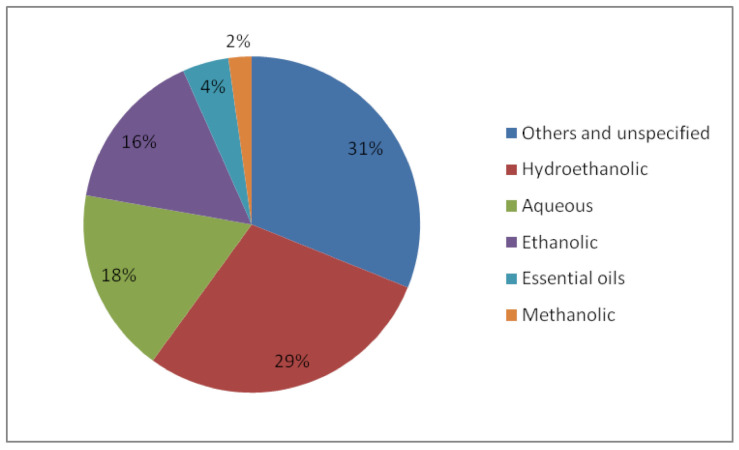
The types of propolis extract used in the included studies.

Moreover, Figure 3 illustrates the types of propolis extracts used in the studies, namely hydroethanolic (29%), ethanolic (16%), aqueous extract (18%), methanolic (2%), propolis essential oils (4%), and unspecified/other forms (31%). These organic solvents are widely used because of their affinity with lipophilic and hydrophilic bioactive molecules, such as phenolics; among them, ethanol is the preferred one because it is non-toxic, economical, and reusable [79].

Table 3 illustrates the geographic locations of the propolis sources and bioactive compounds where whole propolis was not used in the studies; Egypt (6%), Algeria (1%), Cameroon (1%), Morocco (1%), Turkey (14%), Iran (7%), Poland (1%), Korea (1%), India (3%), Indonesia (1%), Malaysia (1%), Brazil (10%), unspecified (19%), and other bioactive compounds (30%). The percentages are rounded to the nearest whole number.

## 4. Discussion

The largest body of experimental evidence found in the present scoping review was in the detoxification theme. The therapeutic properties of propolis and its bioactive compounds appear to be due to their anti-inflammatory properties. In animals and cell cultures which were subjected to chemical and radiation toxicity, propolis was consistently demonstrated to reduce the expression of inflammatory and oxidative markers such as malonaldehyde (MDA), tumor necrosis factor-α (TNF-α), nitric oxide (NO), and inducible nitric oxide synthase (iNOS), while increasing and maintaining antioxidant parameters, namely superoxide dismutase (SOD), glutathione peroxidase (GPx), glutathione reductase (GR), and glutathione (GSH) [23,27,29,30,31,32,34,36]. In addition, it inhibited apoptosis by reducing the expression of genes associated with apoptosis signaling pathways; protein-coding gene *Bax*, *cytochrome*-*c*, *cas*-*3*, *cas*-*8*, and *p53* genes [24,25]. It was also evident that propolis protected cell membranes and prevented further deterioration of the tissue morphology associated with toxicity [23,26,28,33,35,37].

The neuroprotective effect of propolis was also demonstrated in terms of alleviating symptoms associated with aneurysm, ischemia, ischemia-reperfusion and traumatic brain injuries. The anti-inflammatory properties of propolis were shown to play a significant role in attenuating the negative effect of these injuries. Propolis reduced the expression of interleukin-6 (IL-6), TNF-α, matrix metalloproteinase-2 (MMP-2), MMP-9, monocyte chemotactic protein-1 (MCP-1), and iNOS, while increasing the expression of protective proteins such as heat shock protein-70 (hsp70) [14,16,17,18,20]. It also inhibited the development of histopathology associated with these injuries and in some cases promoted the development of myelinated fibers [15,17,21]. More importantly, propolis was shown to significantly ameliorate the impairment of sensory–motor and other physical indices in animals subjected to these injuries [15,18,21,22].

Unsurprisingly, propolis was shown to be effective in attenuating symptoms of neuroinflammation, pain, and oxidative stress. Propolis was consistently shown to reduce inflammation markers such as vascular cell adhesion molecule-1 (VCAM-1), nuclear factor kappa B (NF-kB), mitogen-activated protein kinase (MAPK), and c-Jun N-terminal kinase (JNK)-associated markers in artificially induced inflammation in both cell cultures and animal models. It also reduced the expression of reactive oxygen species (ROS) and pro-inflammatory cytokines such as IL-1β, IL-6, and TNF-α [63,64,66,67,68]. In one study, propolis was shown to prevent the migration of leukocytes into the inflammation site [65]. Propolis also appeared to upregulate the expression of zinc-finger protein A20 during inflammation; a novel anti-inflammatory mode of action of propolis [61].

Moreover, the anti-depressant properties of propolis were demonstrated in various animal model studies. Propolis reduced the level of corticosterone and adenocorticotropic hormones in stressed and depressed animals [71,74]. Apoptosis of neurons in the brain regions such as hippocampus and prefrontal cortex was also inhibited by propolis [72,73]. In addition, it modulated the expression of inflammatory markers such as TNF-α, IL-1β, IL-6, kynurenine (KYN) levels, indoleamine-2,3-dioxygenase activity,5-hydroxytryptamine (5-HT), brain-derived neurotrophic factor (BDNF), and glucocorticoid receptors [73,74,75,76]. The modulation of the endocrines and biochemical markers resulted in the attenuation of depressive behavior and cognitive impairment in the animals.

In the neurological and neurodegenerative disease models, namely Alzheimer’s disease, Parkinson’s disease, and epilepsy, propolis also showed potential therapeutic benefits. Propolis was demonstrated to reduce amyloid fibrillation and reduce the impact of amyloid accumulation [39,42]. In addition, propolis inhibited the activity of both acetylcholinesterase and butyrylcholinesterase in a dose-dependent manner [40,41,42,43]. In the Parkinson’s disease and epilepsy models, propolis reduced neuronal loss and improved the histopathology associated with these diseases [46,47,49,69,70]. In all of these disease models, propolis consistently reduced the expression of inflammatory markers, maintained the antioxidant status, and improved motor/cognitive scores of the animals [44,45,48,69].

One study showed that propolis could potentially be used as an adjuvant for treating brain infection [13]. Nosratiyan et al. (2021) and Shao et al. (2021) demonstrated that propolis can be used in regenerative medicine as it induced axon myelination and oligodendrocyte progenitor cells (OPC) differentiation. Propolis was also shown to be cytotoxic towards cancerous brain cells, i.e., glioblastoma cells, astrocytes, and astroglial cells [50,51,52,54]. However, Kalia et al. (2014) observed no cytotoxicity in organs, including the brain of normal mice fed up to 1000 mg propolis extract/ kg body weight.

**Figure 4:**
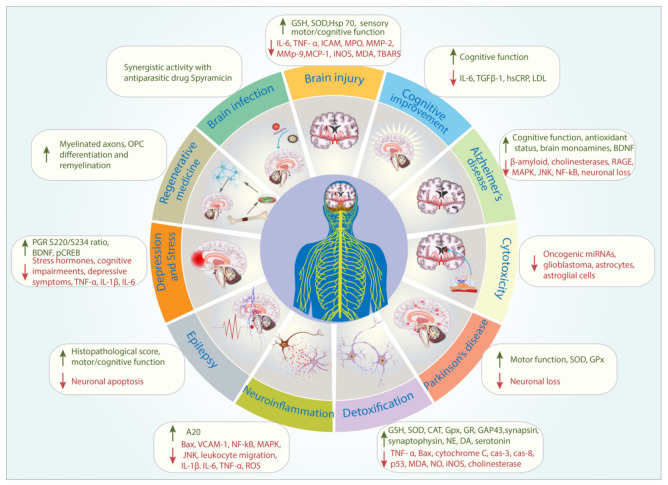
The potential use of propolis in treating brain and neurological injuries and disorders and its mode of action based on the latest experimental data. Green and red arrows indicate increased and reduced activity/expression, respectively.

Arguably, the most important studies were the translation of the therapeutic benefits of propolis into humans demonstrated in randomized, placebo-controlled clinical trials (RCTs). The present scoping review managed to identify three RCTs (*n* = 246 subjects in total). Propolis was shown in all of these studies to improve cognitive function of geriatric subjects measured by various standardized tests; Cognitrax, MMSE, and ADAS-cog. The cognitive improvement appeared to be correlated to the improvement of the serum level of inflammatory markers such as IL-6, TGFβ1, hs-CRP, and serum level of other biochemical markers namely total cholesterol, LDL, urea, creatinine, and uric acid. No adverse event was recorded in these studies [55,57,58]. However, we identified a case report where propolis appeared to induce psychotic episodes in a thirty-four-year-old male in Turkey [77]. Based on various human clinical trials in other areas of propolis research, propolis appears to be generally safe in humans [9,80,81,82,83,84]. Figure 4 summarizes the potential use of propolis as an adjuvant therapy in brain and neurological disorders and injuries.

In the present review, the reviewers adopted a comprehensive and systematic search strategy in order to objectively fulfill the aim of the study. A broad range of studies from all fields of science and technology was collected and analyzed. The reviewers limited the search to studies that were published in the last 10 years, to provide coverage of the latest experimental evidence in the field. However, the reviewers only assessed and included English language articles, which could potentially lead to missing studies from non-English databases, as it is apparent most studies originated from non-English speaking countries. The reviewers also did not assess the quality of the included studies in order to include as many studies and to provide as broad coverage as possible. In addition, the reviewers did not perform meta-analysis as it is not appropriate due to the heterogeneity of the included studies.

## 5. Future Directions and Concluding Remarks

One of the main criticisms often aimed at natural product research is the lack of characterization of the main bioactive compounds. This can be found in the included studies as 31% of the studies did not provide clear identification of the types of propolis extract used. In addition, 19% of the studies did not indicate the geographical location of the propolis source. We propose that all future studies investigating the biological activities of propolis should include at least two pieces of information, namely the types of extract and clear geographical location of the propolis source. Basic chemical analyses, where possible, of the propolis extract, such as total phenolics and/or flavonoids, should be performed. These steps would standardize propolis research, significantly assist in replication studies, and further solidify the potential therapeutic uses of propolis. 

In addition, studies that investigated the biological activities of propolis may refrain from concluding certain phenolics, flavonoids, or terpenoids that impart its biological activities, unless it was clearly demonstrated. It appears the therapeutic benefit of propolis may lie in the synergistic effect of various compounds rather than individual compounds [65]. Furthermore, the majority of propolis extracts used were extracted using organic solvents such as ethanol and methanol. Concerns with regard to chemical toxicity, contamination, religious and cultural reasons often arise due to the use of organic solvents for extraction purposes. Another promising area of research is the use of safer and greener chemical alternatives such as natural deep eutectic solvents (NADES), glycerol, and propylene glycol [85,86,87]. Moreover, the majority of the studies used propolis from a single species of bees; the European honey bee (*Apis mellifera*). Future research could also explore the potential therapeutic properties of propolis harvested from the hives of other bee species such as *Apis cerana* and *Meliponini* bees (stingless bees). In conclusion, the present scoping review demonstrates that propolis is a promising therapeutic substance, either as pharmaceuticals or nutraceuticals, for protecting the brain and treating neurological disorders and injuries.

## Figures and Tables

**Table 1:** The key themes of the included studies.

Themes	Percentage (%)
Adverse effects	2
Alzheimer’s disease model	9
Brain infection	2
Cognitive Improvement	6
Cytotoxicity	8
Depression and Stress models	9
Detoxification	24
Epilepsy model	3
Ischemia/Ischemia-Reperfusion injury/Traumatic Brain Injury/Aneurysm	14
Neuroinflammation, Pain, and Oxidative stress	12
Parkinson’s disease model	8
Regenerative medicine	3
Others	2

**Table 2:** The summary of the included studies in the present scoping review.

Themes	References	Types of Study	Types of Propolis Extracts and/or Propolis Bioactive Compounds	Geographic Locations of the Propolis Source	Measured Outcome
Brain Infection	[13]	Animal model (*n* = 140, rats)	Hydroethanolic extract 0.1 mL of 1:10 *w/v* (25 g in 250 mL)	Egypt	Propolis enhanced the anti-*Toxoplasma gondii* activity of Spiramycin
Ischemia/Ischaemia-Reperfusion injury/ Traumatic Brain Injury/Aneurysm	[14]	Animal model (*n* = 12, rabbit)	Ethanolic extract 100 mg/kg BW	Turkey	Neuroprotective properties of propolis demonstrated in the post-operative Tarlov scores, biochemical parameters such as interleukin-6 (IL-6), tumor necrosis factor (TNF)-α, myeloperoxidase activity, ischemia-modified albumin (IMA), intercellular adhesion molecule-1 (ICAM-1), total oxidant status, and histopathological examination.
	[15]	Animal model (*n* = 72, mice)	Aqueous extract 30, 100 and 200 mg/kg BW	Iran	Propolis improved grasping ability and sensory–motor function following permanent middle cerebral artery occlusion.
	[16]	Animal model (rats, *n* = 33)	Hydroethanolic extract 200 mg/kg BW	Indonesia	Propolis induced the expression of the protective heat-shock protein (hsp)-70 and reduced the expression of inflammatory markers such as caspase-3 and apoptosis inducing factor in traumatic brain injury animal model.
	[17]	Animal model (rats, *n* = 36)	Caffeic acid phenethyl ester (CAPE) 10 µmol	Not specified	CAPE reduced the severity of elastase-induced aortic aneurysm by reducing the expression of metalloproteinases MMP-2 and MMP-9, monocyte chemotactic protein-1 (MCP-1), and inducible nitric oxide synthase (iNOS). CAPE also circumvented the loss of vascular smooth muscle cells (VSMCs) in aortic walls of treated rats.
	[18]	Animal model (mice, *n* = 72)	Aqueous extract 100 and 200 mg/kg BW	Iran	In cerebral ischemia-induced mice, propolis reduced the expression of malondialdehyde (MDA) and improved the antioxidant status (ratio of superoxide dismutase (SOD) to glutathione peroxidase GPx). Propolis also ameliorated the sensory–motor impairment and neurological deficits induced by cerebral ischemia.
	[19]	Cell culture (neuroblastoma N2)	Hydroethanolic extract	Not specified	Propolis reduced the extent of neuronal damage in oxygen-glucose deprivation/reoxygenation (OGD/R)- induced cells.
	[20]	Animal model (mice, *n* = 40)	Chrysin 50 mg/kg BW	Not specified	Chrysin improved glutathione (GSH) level and decreased thiobarbituric acid reactive substances (TBARS) level. Chrysin attenuated the development of neurodegenerative histopathologies associated with global cerebral ischemia/reperfusion in mice.
	[21]	Animal model (rats, *n* = 36)	Hydroethanolic extract1 and 10 mg/kg BW	Brazil	In sciatic nerve-injured rats, propolis improved the motor function and sciatic functional index. Propolis also significantly accelerated the motor recovery and increased the number of myelinated fibers.
	[22]	Animal model (rats, *n* =75)	Propolis powder dissolved in water 200-mg/kg BW	Turkey	Propolis improved motor function (walking track and electrophysiological analyses), following crush injury of the sciatic nerve in rats.
Detoxification	[23]	Cell cultures (SH-SY5Y)	Pinocembrin 1–25 µM	Not specified	Pinocembrin prevented the Chlorpyrifos-induced mitochondrial membrane potential (MMP) loss and ATP synthesis inhibition in SH-SY5Y cells. Pinocembrin also induced the anti-inflammatory activity.
	[24]	Cell cultures (SH-SY5Y)	Caffeic acid, chrysin, quercetin and ferulic acid 100, 200 and 400 µM	Not specified	The phenolic compounds inhibited the cyclophosphamide-induced apoptosis of SH-SY5Y cells.
	[25]	Cell cultures (SH-SY5Y)	Chrysin 0.05 mM	Not specified	Chrysin inhibited the diclofenac-induced apoptosis of SH-SY5Y cells. Chrysin inhibited the expression of *Bax*, *cytochrome c*, *cas*-3, *cas*-8 and *p53* genes associated with diclofenac treatment.
	[26]	Animal model (rats, *n* = 40)	Hydroethanolic extract 200 mL/kg BW	Egypt	Propolis reduced the development of aluminum silicate-induced irregular euchromatic nucleus and significantly increased the invagination of the nuclear envelope of Purkinje cells in the cerebellar cortex of aluminum silicate-intoxicated rats.
	[27]	Animal model (rats, *n* = 24–32)	Not specified 600 mg/kg BW	Not specified	Propolis reduced the expression of inflammatory markers; malondealdehyde (MDA) and nitric oxide (NO). Propolis improved the antioxidant status by maintaining glutathione level and the activity of superoxide dismutase and catalase in monosodium glutamate-intoxicated rats. Propolis also prevented the accumulation of β-amyloid and oxidative-stress marker 8-hydroxy-2′-deoxyguanosine.
	[28]	Animal model (rats, *n* = 24)	Propolis capsules (extract not specified) 50 mg/kg BW	Not specified	Propolis attenuated the Paclitaxel-induced morphological deterioration of myelinated fibers of sciatic nerve.
	[29]	Animal model (rats, *n* = 60)	Not specified 100 and 200 mg/kg	Not specified	Propolis reduced the expression of inducible nitric oxide synthase (iNOS) gene in thioacetamide (TAA)-induced rats.
	[30]	Animal model (rats, *n* = 120)	Hydroethanolic extract 200 mg/kg BW	Egypt	Propolis reduced the adverse effect of methotrexate by reducing MDA and increasing the activity of antioxidant enzymes such as superoxide dismutase (SOD), glutathione peroxidase (GPx), and glutathione reductase (GR), and GSH.
	[31]	Animal model (rats, *n* = 40)	Propolis (extract not specified) and CAPE	Not specified	Propolis and CAPE prevented the increase in xanthine oxidase activity, nitric oxide synthase activity, nitric oxide (NO●) and peroxynitrite (ONOO−) levels in radiation-treated rats.
	[32]	Animal model (rats, *n* = 24)	Chrysin 50mg/kg BW	Not specified	In 3-Nitropropionic acid treated rats, chrysin improved the behavioral performance and attenuated the oxidative stress by maintaining the level of antioxidant parameters and reducing the oxidative stress parameters.
	[33]	Animal model (rats, *n* = not specified)	Aqeuous extract 100 mg/kg BW	Turkey	Propolis reversed the scopolamine-induced cognitive deterioration.
	[34]	Animal model (rats, *n* = 54)	Propolis (extract not specified) 80 mg/kg BW and CAPE 10 µmol/kg BW	Not specified	Propolis prevented the increase in MDA associated with radiation toxicity.
	[35]	Cell cultures (PC-12)	CAPE 1, 5 or 10μM	Not specified	CAPE induced the formation of synapses and neuritis, and prevented the MPP+ (1-methyl-4-phenylpyridinium) cytotoxicity by increasing the expression of increases the expression of GAP-43, synapsin and synaptophysin.
	[36]	Animal model (rats, *n* = 24)	Hydroethanolic 150 mg/kg BW	Malaysia	Propolis inhibited the expression of NOS, NO, TNF-α and caspase-3 in the cerebral cortex (CC), cerebellum (CB) and brain stem (BS) of kainic acid-induced rats.
	[37]	Animal model (rats, *n* = 40)	Not specified 50 mg/kg BW	Not specified	Propolis attenuated chlorpyrifos-induced toxicity. Propolis reduced the activity of serum and brain cholinesterase induced by chlorpyrifos. Propolis also inhibited the increase in glial fibrillary acidic protein-expression.
	[38]	Animal model (rats, *n* = 78)	Not specified 150mg/kg BW	Egypt	In endotoxin-treated rats, propolis attenuated the decrease in the level of norepinehrine (NE), dopamine (DA) and 5-hydroxytryptamine (serotonin, 5-HT) in both thalamus-hypothalamus and cerebellum.
Alzheimer’s disease model	[39]	In vitro	Hydroethanolic extract 1:10 (*w/v*)	Iran	Propolis inhibited amyloid fibrillation.
	[40]	In vitro	Essential oils of propolis	Cameroon	Components of propolis essential oil exhibited anti-cholinesterase activity.
	[41]	In vitro	Essential oils and methanolic extract IC_50_ value: 20–35 μg/mL	Algeria	Components of propolis essential oil and methanolic extract of propolis exhibited anti-cholinesterase activity against both acetylcholinesterase and butyrylcholinesterase.
	[42]	Animal model (rats, *n* = 56)	Ethanolic extract 100, 200, 300 mg/kg BW	India	Propolis reduced the severity of the cognitive impairment in the β-amyloid induced rats. Propolis improved the antioxidant status, brain monoamines, and brain-derived neurotrophic factor. Propolis inhibited the activity of acetylcholinesterase activity in a dose-dependent manner.
	[43]	In vitro	CAPE Ki = 322.02 pM to 4.467 μM	Not specified	Inhibition of the activity of acetylcholinesterase and butyrylcholinesterase.
	[44]	Animal model (mice, *n* = 48)	Pinocembrin 20 and 40 mg/kg BW	Not specified	Pinocembrin inhibited the expression of receptor for advanced glycation end-products (RAGE) and its downstream inflammatory signaling pathway markers such as p38 mitogen-activated protein kinase (MAPK), protein kinase (SAPK)/c-Jun N-terminal kinase (JNK), and NF-κB. Pinocembrin also exhibited mitochondrial-protective properties.
Parkinson’s disease model	[45]	Animal model (*Drosophila melanogaster*, *n* = not specified)	Ethanolic extract 250 and 500 mg/mL	Not specified	Propolis improved motor activity, antioxidant status, and lifespan.
	[46]	Animal model (rats, *n* = 21)	Not specified 200 mg/kg BW	Brazil	Propolis inhibited neuronal loss in the substantia nigra and attenuated striatal fiber degeneration in 6-hydroxydopamine (6-OHDA)-induced rats.
	[47]	Animal model (rats, *n* = 48)	Hydroethanolic extract of propolis (10 and 50 mg/kg BW) and Formononetin (10 and 20 mg/kg BW)	Brazil	Propolis and one of its bioactive compound, formononetin reduced the neuron loss and motor impairment in 6-OHDA-induced rats.
	[48]	Animal model (rats, *n* = 70)	Aqueous extract 1:5 (*w/v*)	Iran	Propolis improved the antioxidant status in terms of SOD and GPx activities and ferric reducing ability of plasma in 6-OHDA-induced rats. Propolis also appeared to protect tyrosine hydroxylase neurons.
	[49]	Animal model (rats, *n* = 18)	CAPE 10μmol/kg BW	Not determined	CAPE prevented the dopaminergic neuronal loss induced by 6-OHDA in rats. CAPE also prevented mitochondrial permeability transition (neuronal death mediator)
Cytotoxicity	[50]	Cell cultures (glioblastoma)	Hydroethanolic extract	Turkey	Propolis reduced the expression of oncogenic miRNAs associated with glioblastoma.
	[51]	Cell cultures (glioblastoma)	Propolis extracted in Lavender oil 1:10 (*w/v*)	Turkey	Cytotoxic activity against glioblastoma cells.
	[52]	Cell cultures (rat primary astrocytes)	Hydroethanolic extract 10, 25, or 100 µg/ml	Turkey	Dose-dependent cytotoxicity on astrocytes was observed. Propolis inducedcytoskeletonrearrangements and pro-apoptotic signaling pathways; NF-kB and poly (ADP-ribose) polymerase (PARP).
	[53]	Animal model (mice, *n* = not specified)	Ethanolic extract up to 1000 mg/kg BW	India	High concentration of propolis extract up to 1000 mg/kg BW did not negatively affect the histological appearance of organs, including the brain.
	[54]	Cell cultures (astroglia cell line/SVGp12)	Ethanolic extract 10–100 µg/mL CAPE and Chrysin 5–50 µM	Poland	Propolis and its bioactive compounds reduced the viability of astroglial cells.
Cognitive Improvement	[55]	Randomized, placebo-controlled trial (*n* = 79)	Dietary supplement containing propolis extract. Types of extract not specified 6 capsules of propolis extract containing artepillin C, 57.68 mg; culifolin, 0.95 mg	Not specified	Propolis significantly improved verbal memory and information processing speed (Cognitrax). Propolis also improved serum total cholesterol, LDL cholesterol, urea nitrogen, creatinine, and uric acid.
	[56]	Animal model (rats, *n* = 40)	Aqueous extract 100 mg/kg BW	Turkey	Propolis reversed the transfer latency parameter associated with physiological aging in rats. Transfer latency is defined as the time taken by the animals to move from the open arm to the enclosed arm of an experimental compartment.
	[57]	Randomized, placebo-controlled trial (*n* = 80)	Propolis dietary supplement (types of extract not specified) 0.33 g	Brazil	Propolis improved cognitive function measured by Mini-Mental State Examination (MMSE) and Alzheimer Disease Assessment Scale-cognitive subscale (ADAS-cog). Propolis reduced serum level of IL-6 and TGFβ1.
	[58]	Randomized, placebo-controlled trial (*n* = 87)	Propolis dietary supplement (Extract not specified)	Brazil	Propolis improved cognitive function measured by MMSE and reduced the serum level of hs-CRP and LDL.
Regenerative medicine	[59]	Animal model (mice, *n* = 24)	Implantation of an artificial guidance channel containing whole propolis combined with Gum Arabic.	Iran	Propolis–gum Arabic graft increased the mean number of muscle fiber diameters and myelinated axons.
	[60]	Oligodendrocyte progenitor cell (OPC) cultures Animal model (mice, *n* = 30)	Pinocembrin 10 mM	Not specified	Pinocembrin induced the OPC differentiation and remyelination through the phosphorylated mTOR pathway in multiple sclerosis disease model.
Neuroinflammation, Pain, and Oxidative stress	[61]	Cell cultures (BV2 cells and primary microglia cells) Animal model (mice, *n* = 16)	Chrysin 5, 10, and 20 µM	Not specified	Chrysin inhibited the inflammation of LPS-induced BV2, primary microglial cells, and mice by upregulating the expression of zinc-finger protein A20.
	[62]	Animal model (rats, *n* = 18)	Aqueous extract 100 and 200 mg/kg BW	Not specified	Propolis decreased the expression of *Bax* and reduced the number of neurons in the hippocampal CA1 area of sodium nitrite-induced rats.
	[63]	Cell cultures (bEnd.3)	Chrysin 10, 30, and 100 µM	Not specified	Chrysin reduced the expression of vascular cell adhesion molecule-1 (VCAM-1), Nuclear factor-κB (NF-κB), p38 mitogen-activated protein kinase (MAPK), and c-Jun N-terminal kinase in the LPS-induced bEnd.3 cells. Chrysin also prevented the adhesion of monocytes to the LPS-induced bEnd.3 cells.
	[64]	Cell culture (microglia) and animal model (mice, *n* = not specified)	Not specified 50 μg/mL	Brazil	Propolis inhibit the cytotoxicity and the expression of pro-inflammatory biomarkers; IL-1β, TNF-α, IL-6, and 8-oxo-deoxyguanosine following hypoxia exposure. Propolis also significantly reduced the hypoxia-induced generation of reactive oxygen species (ROS) in the mitochondria and downregulated the expression of nuclear factor-κB (NF-κB) in microglia.
	[65]	Animal model (mice, *n* = 180; rats, *n* = 36)	Hydroethanolic extract of propolis 3, 10, and 30 mg/kg BW and formononetin 10 mg/kg BW	Brazil	Propolis and formononetin demonstrated anti-inflammatory activity. Propolis and formononetin inhibited oedema response and carrageenan-induced leukocyte migration during inflammatory process. Propolis was also shown to have antinoceptive properties on inflammatory and neurogenic pain.
	[66]	Animal model (rats and mice, *n* = 20)	Aqueous extract 1, 2.5, abd 5% (*w/v*)	Morocco	Propolis exhibited both central and peripheral antinociceptive activities.
	[67]	Cell cultures (microglia) and mice, *n* = 9)	Ethanolic extract 5, 50, and 500 µg/mL	Brazil	Propolis reduced the expression of oxidative markers IL-1*β*, TNF-*α*, IL-6, and 8-oxo-deoxyguanosine. Propolis also reduced the production of ROS in mitochondria.
	[68]	Animal model (mice, *n* = 36)	Ethanolic extract 1 mM	Brazil	Propolis exhibited antinoceptive properties by modulating the expression of IL-1β and TNF-α.
Epilepsy model	[69]	Animal model (rats, *n* = 50)	Aqueous extract 0.012 mg/kg BW	Turkey	Propolis increased the histopathological score of the hippocampus and motor/cognitive score in the lithium and pilocarpine-induced rats.
	[70]	Animal model (rats, *n* = 21)	CAPE 30 mg/kg BW	Not determined	CAPE significantly protected the number of neurons in the CA1, CA3, and dentate gyrus regions of the hippocampus and the prefrontal cortex. Apoptosis in the hippocampus and the prefrontal cortex was also inhibited by CAPE.
Depression and Stress models	[71]	Animal model (rats, *n* = 40)	Aqueous extract 100 mg/kg BW	Turkey	In chronic unpredictable mild stress (CUMS)-induced rats, propolis exhibited anti-depressant properties. Propolis also reduced the level of corticosterone level and reversed cognitive impairments.
	[72]	Animal model (rats, *n* = 24)	Not specified 100 and 200 mg/kg BW	Not specified	Propolis prevented the hippocampal areaneuronal loss associated with stress.
	[73]	Animal model (mice, *n* = 42)	Chrysin 5 and 20 mg/kg BW	Not specified	Chrysin reduced the elevation of corticotropin-releasing and adrenocorticotropic hormones, and tumor necrosis factor-α, interleukin-1β, interleukin-6 and kynurenine levels in the prefrontal cortex (PFC) and hippocampus (HP) in mice exposed to unpredictable chronic stress.
	[74]	Animal model (mice, *n* = 40)	Chrysin 5 or 20 mg/kg BW	Not specified	Chrysin alleviated behavioral modification following olfactory bulbectomy. Chrysin attenuated the alterations of biochemical markers associated with depressive behavior, namely tumor necrosis factor-α, interferon-γ, interleukin-1β, interleukin-6, kynurenine (KYN) levels, indoleamine-2,3-dioxygenase activity,5-hydroxytryptamine (5-HT), brain-derived neurotrophic factor (BDNF), KYN/tryptophan and 5-hydroxyindoleacetic acid/5-HT ratio.
	[75]	Animal model (mice, *n* = 28)	CAPE 5, 10, and 20 μmol/kg	Not specified	CAPE exhibited anti-depressant activity on the animals. CAPE also reduced CAPE significantly decreased glucocorticoid receptor (GR) phosphorylation at S234 (pGR(S234)).
	[76]	Animal model (mice, *n* = 50)	Hydroethanolic extract 50, 100, and 200 mg/kg BW	Korea	Propolis exhibited anti-depressant activity by increasing the expression of hippocampal glucocorticoid receptor. Propolis also increased pGR(S220)/(S234) ratio. Propolis upregulated the cAMP-responsive element binding protein phosphorylation at S133 (pCREB).
Adverse effects	[77]	Case report	Not specified 50 g/day for 3 days	Turkey	Propolis appeared to induce psychotic episodes in a thirty four year old male.
Others	[78]	Cell cultures (rat brain microvascular endothelial cells)	Pinocembrin 5, 20, and 40 µg/mL	Not specified	Pinocembrin appeared to cross blood–brain barrier cell model without affecting the function and expression of p-glycoprotein.

**Table 3:** The percentages of studies that utilize propolis bioactive compounds and/or the geographical locations of the propolis source in the included studies.

Geographical Sources of Propolis and/or Bioactive Compounds	Percentage (%)
Bioactive compounds	30
Unspecified	19
Turkey	14
Brazil	10
Iran	7
Egypt	6
India	3
Algeria	1
Morocco	1
Cameroon	1
Poland	1
Korea	1
Indonesia	1
Malaysia	1

## Supplementary Materials

The following are available online at https://www.mdpi.com/article/10.3390/biomedicines9091227/s1, Table S1: Search strategy of the scoping review, Prisma checklist.
